# Establishment of the Rat Model of Intracranial Aneurysm Contributing to the Development of Endovascular Devices

**DOI:** 10.3390/biomedicines14040810

**Published:** 2026-04-02

**Authors:** Masahiko Itani, Tomohiro Aoki

**Affiliations:** Department of Pharmacology, The Jikei University School of Medicine, 3-25-8 Nishi-Shinbashi, Minato-ku, Tokyo 105-8461, Japan; masa9426@kuhp.kyoto-u.ac.jp

**Keywords:** animal model, coil, endovascular device, intracranial aneurysm, platform, preclinical model, rat

## Abstract

**Background**: Endovascular treatment has become the major choice for treating intracranial aneurysm (IA). The development of novel endovascular devices for IA treatment is, therefore, socially important. For this purpose, a preclinical animal model to test a prototype of devices plays a crucial role. The major problems regarding currently used preclinical animal models, mainly in medium-to-large animals, are the expense and the lack of IA pathology, as they only mimic the morphological aspect. **Methods**: Sprague–Dawley rats were used, and the new bifurcation was formed via end-to-side anastomosis of carotid arteries. An aneurysm lesion induced at the newly formed bifurcation site was macroscopically assessed. Endovascular coiling of the induced aneurysm was then done. **Results**: An aneurysm lesion with a balloon-like shape, as in human cases, was induced at the newly formed bifurcation site. Some of the induced lesions spontaneously ruptured. Endovascular coiling was successfully done by using the micro-catheter and coil used at the clinical site. **Conclusions**: The rat model of IAs established here provides a novel platform contributing to the development of endovascular devices to treat IAs and, therefore, significantly facilitates the development of devices to achieve more effective treatment.

## 1. Introduction

Intracranial aneurysm (IA) has become a major cause of subarachnoid hemorrhage (SAH), with a high mortality and morbidity rate [1,2]. Because of the poor outcome after the onset or recurrence of SAH, some IAs with a higher probability of rupture or those already ruptured are surgically treated to prevent rupture and resultant deterioration. Currently, more and more IAs are endovascularly treated due to the less invasive nature compared with micro-neurosurgical clipping [3]. The development of a novel endovascular device to effectively treat IAs is thus essential for society.

To develop a novel device, a preclinical animal model to validate various aspects required for an endovascular device, such as effectiveness or safety, plays a crucial role. Nowadays, medium-to-large animals are used, most typically pigs or rabbits, and only the morphological aspect of IA lesions is mimicked by a venous pouch or the ligation of an artery, like the vertebral artery, to create a blind end [4,5]. However, such an animal model is costly and fails to reproduce IA pathology and the response of IA walls to a deployed device. 

In addition, most existing models are artificially created and do not reproduce aneurysm formation at arterial bifurcation sites or the spontaneous development process observed in human IAs.

In the present study, to overcome many limitations of the above animal models, we have established a rat model of IAs that reproduces IA progression as observed in human cases.

This model uniquely enables spontaneous aneurysm formation at arterial bifurcation sites and demonstrates histopathological features closely resembling those of human intracranial aneurysms.

Furthermore, prototype devices for human clinical usage can be deployed in this model.

## 2. Materials and Methods

### 2.1. Study Approval of Animal Experiments

All of the following experiments, including animal care and use, complied with the National Institute of Health’s Guide for the Care and Use of Laboratory Animals, followed the ARRIVE guidelines (https://arriveguidelines.org), and were approved by the Institutional Animal Care and Use Committee of the Jikei University School of Medicine (Approval Number; #2023-004).

### 2.2. Induction of Aneurysm in Rats

Nine-week-old male Sprague–Dawley rats were purchased from Japan SLC (Slc: SD, Shizuoka, Japan). Animals were maintained on a light/dark cycle of 12 h/12 h and had free access to chow and water.

To induce aneurysm, under general anesthesia by inhalation of isoflurane (induction; 5.0%, maintenance; 1.5~2.0%, #IYESC-0001, Pfizer Inc., New York, NY, USA), animals were subjected to the cutting of the right common carotid artery and then anastomosed to the left common carotid artery in an end-to-side fashion to create a bifurcation with a 10-0 nylon suture. The left renal artery was also ligated with a 10-0 nylon. All experimental procedures were conducted at room temperature. The carotid artery occlusion time (ischemia time) was approximately 30 min. The suturing was performed using interrupted stitches rather than continuous sutures. Systemic heparinization was not administered during the procedure. Immediately after the above surgical manipulations, animals were fed chow containing 8% sodium chloride and 0.12% 3-aminopropionitrile (Tokyo Chemical Industry, Tokyo, Japan), an inhibitor of lysyl oxidase that catalyzes the cross-linking of collagen and elastin. These procedures were performed in accordance with previously established intracranial aneurysm models [6]. This combination of procedures is based on previously established experimental models of intracranial aneurysms, in which increased hemodynamic stress induced by hypertension and structural weakening of the arterial wall synergistically promote aneurysm formation. Renal artery ligation and a high-salt diet were used to induce hypertension, while 3-aminopropionitrile was administered to inhibit collagen cross-linking and facilitate degeneration of the vascular wall.

Two weeks after the above surgical manipulations, animals were subjected to digital subtraction angiography and endovascular coiling as described in the following section. We selected 14 days as the observation endpoint based on our previous experimental experience, in which aneurysm formation was consistently observed at approximately two weeks after the surgical procedures [7].

### 2.3. Digital Subtraction Angiography and Endovascular Coiling

Digital Subtraction Angiography was done by using X-ray fluoroscopy (#Artis zee, Siemens, Munich, Germany) via the tail artery or the femoral artery under general anesthesia by inhalation of isoflurane (induction; 5.0%, maintenance; 1.5~2.0%, #IYESC-0001, Pfizer Inc.).

For the angiography technique in rats, we incised the skin of the tail. Then we were able to see the tail artery, clip it, and cut down the artery wall. After that, we directly inserted the microcatheter (Excelsior SL-10, Stryker, Kalamazoo, MI, USA). The microcatheter was advanced into the left CCA under fluoroscopy guidance. Once in position, the guidewire was withdrawn. A 0.5 mL bolus of contrast agent (Iohexol, 300 mg iodine/mL; Daiichi Sankyo, Tokyo, Japan) mixed with heparin (1 mg Iohexol: 10 units heparin) was injected rapidly. The platinum coil (Target 360, Stryker, USA) was then deployed by using the microcatheter.

## 3. Results

### 3.1. Induction of Aneurysm Lesion at the Surgically Created Bifurcation in Carotid Artery of Rats

**Figure 1:**
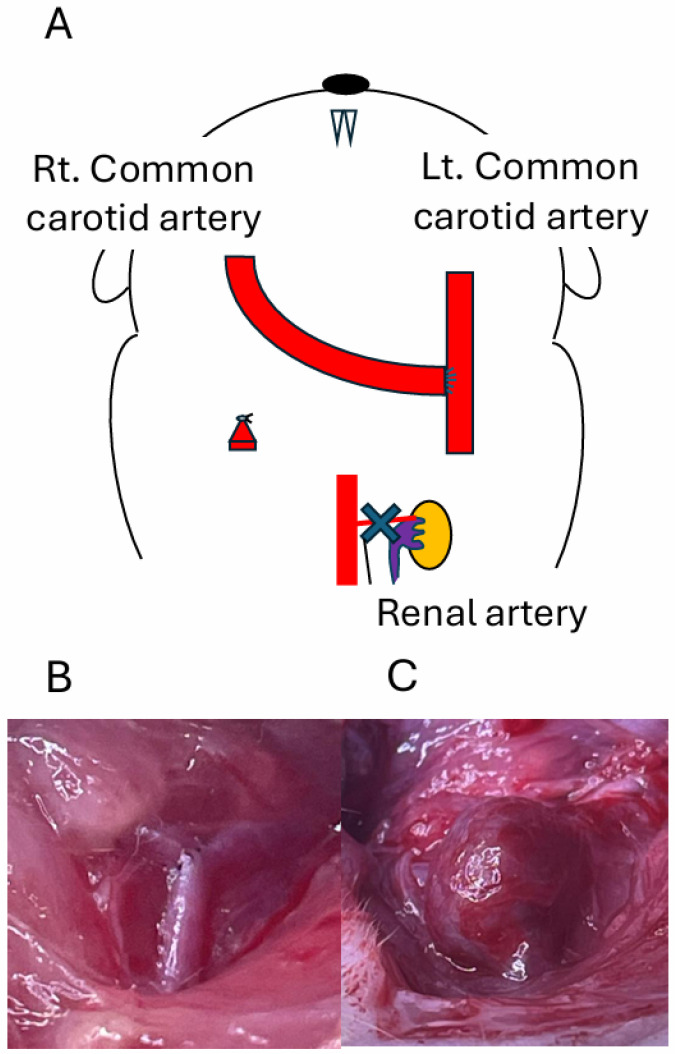
Induction of an aneurysm lesion at the newly formed carotid bifurcation in rats. The bifurcation was created at the cervical portion of rats through end-to-side anastomosis of common carotid arteries, as in the schematic drawing (**A**). The macroscopic images of the newly formed bifurcation site before (**B**) and after (**C**) the induction of saccular aneurysm lesion are shown.

**Figure 2:**
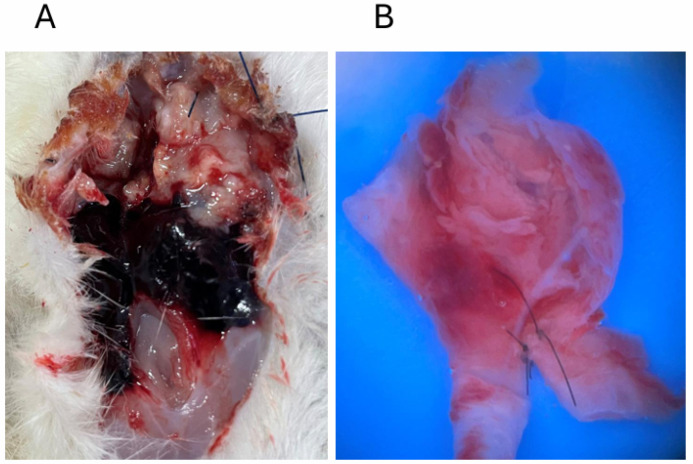
Rupture of induced aneurysms in rats. The macroscopic images of hemorrhage due to the rupture of an induced aneurysm (**A**) and ruptured aneurysm lesion induced at the newly formed bifurcation site (**B**) are shown.

The bifurcation site was created by the end-to-side anastomosis of common carotid arteries (Figure 1A). Aneurysm lesions with a balloon-like shape were induced at the newly formed bifurcation at about 14 days after the surgical manipulation (Figure 1B,C). Induced aneurysms sometimes spontaneously ruptured (Figure 2), like IAs in human cases (rupture was observed in 2 out of 12 rats). We used 12 rats for this model, and nine aneurysms were detected on the angiography. The remaining three rats did not develop aneurysms.

### 3.2. Histology of the Aneurysm in This Model

**Figure 3:**
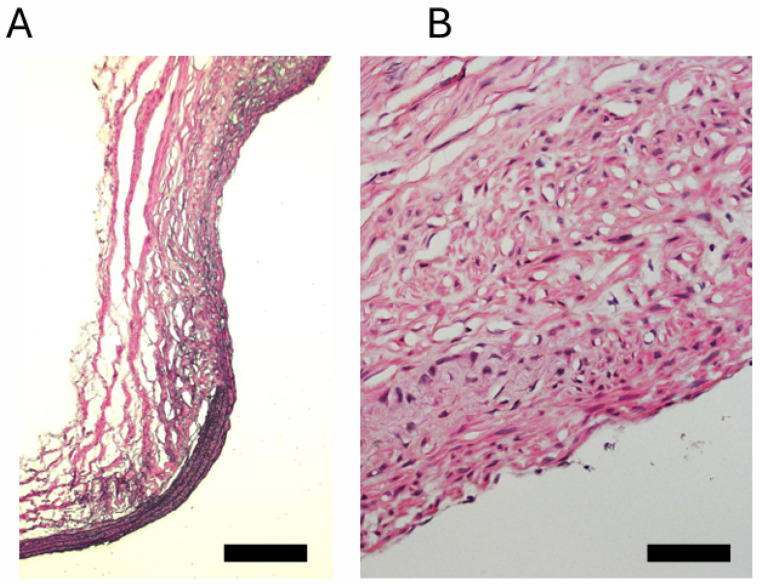
Histology of aneurysm in this model. Representative histopathological images of this model are presented. Consistent with human intracranial aneurysms, the lesions exhibited disruption of the internal elastic lamina, thinning of the medial layer, and inflammatory cell infiltration on the adventitial side. (**A**) scale bar:100 μm (**B**) scale bar: 20 μm.

Histopathological analysis revealed disruption of the internal elastic lamina, accompanied by thinning of the medial layer. The smooth muscle cell layer appeared sparse and disorganized, and inflammatory cell infiltration was observed predominantly in the adventitial side of the aneurysm wall (Figure 3).

### 3.3. Endovascular Coiling of Induced Aneurysm in Rats

**Figure 4:**
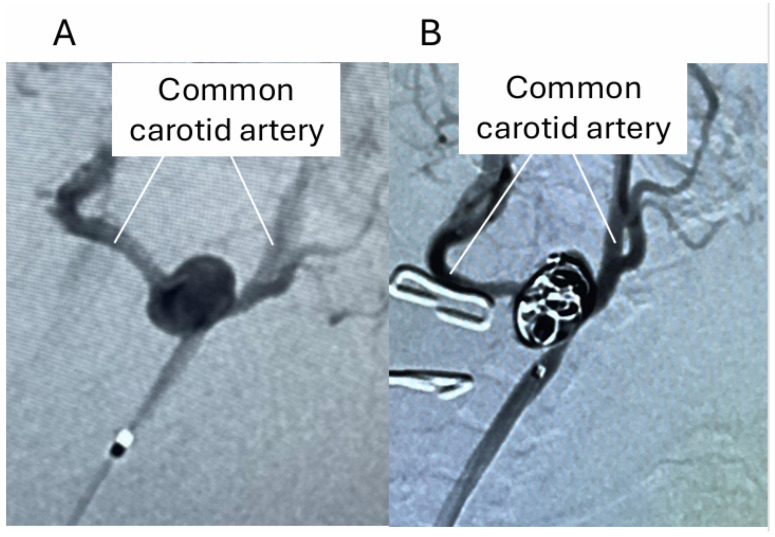
Endovascular coiling of induced aneurysms in rats. The images from digital subtraction angiography before (**A**) and after (**B**) coil embolization of the induced aneurysm are shown.

Endovascular coiling of the induced aneurysm lesion at the newly formed bifurcation of the carotid arteries was successfully done by using a micro-catheter and coil used at the clinical site (Figure 4), as in human cases.

### 3.4. Result of All Procedures

Aneurysm formation was observed in 9 out of 12 rats (75%). Among these, spontaneous ruptures occurred in two cases. In addition, one aneurysm ruptured after coil embolization. Endovascular access using a microcatheter was successfully achieved in all animals, and coil embolization was successfully performed in all aneurysm-bearing cases (9/9). The remaining two rats did not develop aneurysms.

## 4. Discussion

We have developed the rat model of IAs that can reproduce the IA progression of human cases and allows endovascular coiling by using coils for clinical usage. The rat model of IAs we have developed has considerable merit compared with the widely used models in medium-to-large animals. The rat model has cost-effectiveness; therefore, various prototypes of devices can be easily tested. Also, this kind of model can contribute to clarifying the response of IA walls to deployed devices that are not examined in other currently used models. Thereby, the established rat model of IAs can contribute to the development of a novel endovascular device and will thus improve treatment outcome.

Furthermore, this model has additional advantages in that aneurysms are formed spontaneously at arterial bifurcation sites and exhibit histopathological features closely resembling those of human intracranial aneurysms. Although pseudoaneurysm formation cannot be completely excluded, the histopathological findings in this study suggest that the lesion is distinct from a pseudoaneurysm and resembles human intracranial aneurysms.

Through the recent advancement of experimental techniques to establish gene-modified rats, especially by the CRISPR-Cas9 system [8,9,10,11], various kinds of gene-modified rat strains can be applied to the established model. Such a model using gene-modified animals makes it possible to conduct experiments to analyze molecular machineries regulating the healing process after the deployment of a device, or to monitor the response of a specific type of cell. In this respect, the present model has the potential to provide additional experimental opportunities compared with conventional models.

## 5. Imitation

This study has several limitations. First, the model allows the use of only small-diameter microcatheters, which may restrict the applicability of certain endovascular devices. Second, the induced aneurysms are formed in extracranial vessels rather than intracranial arteries, which may limit the extent to which this model fully replicates the anatomical and physiological conditions of human intracranial aneurysms. Third, the sample size was limited and the follow-up period was relatively short; therefore, further validation is required.

## 6. Conclusions

To our knowledge, this study presents a rat model of intracranial aneurysm that enables endovascular coil embolization using clinically relevant devices.

This model should be considered as a proof-of-concept and may serve as a platform for preliminary evaluation of endovascular devices, taking advantage of the benefits of small animal models. Further studies are required to validate its translational applicability.

## Data Availability

The original contributions presented in this study are included in the article. Further inquiries can be directed to the corresponding author.
